# Sex-specific mechanisms for eating disorder risk in men and women with autistic traits: the role of alexithymia

**DOI:** 10.1186/s40337-023-00746-7

**Published:** 2023-02-10

**Authors:** R. L. Moseley, C. Atkinson, R. Surman, M. Greville-Harris, L. May, L. Vuillier

**Affiliations:** 1 Department of Psychology, Bournemouth University, Poole, UK; 2 Dorset Healthcare University NHS Foundation Trust, Poole, UK

**Keywords:** Eating disorders, Alexithymia, Anxiety, Depression, Autistic traits, Autism, Sex differences

## Abstract

**Background:**

A poorly understood relationship exists between eating disorders (ED) and autism spectrum conditions (ASC: henceforth ‘autism’). ED are more prevalent in autistic people and people with high autistic traits, and autistic features are prognostic of longer illness. Aiming to understand what increases the risk of ED in relation to autism and autistic traits, previous research has implicated alexithymia as a causal mechanism in this relationship. These studies could not, however, disentangle whether alexithymia explains the relationship between ED pathology and autistic traits directly or through its impact on anxious/depressive symptoms, which in turn result in higher ED symptomatology. Moreover, despite evidence for sex differences in the aetiology of ED, little research has examined the impact of sex on these relationships.

**Methods:**

Focusing on the association between autistic traits and ED psychopathology, we examined *independent* mediating effects of alexithymia and anxious/depressive symptoms, as well as *sequential* mediation effects where alexithymia affects ED psychopathology via its impact on anxious/depressive symptoms. Participants were 198 men and 265 women with formally diagnosed and suspected ED, who completed an online survey of standardised scales.

**Results:**

In men, higher autistic traits were associated with ED psychopathology sequentially via greater alexithymia and through that, greater depressive/anxious symptoms. In women, alexithymia mediated the relationship between autistic traits and ED psychopathology both directly and sequentially through its impact on anxious/depressive symptoms. Interestingly, depressive/anxious symptoms also mediated that relationship independently from alexithymia.

**Conclusions:**

While cross-sectional, these findings suggest that the relationship between autistic traits and ED symptomatology is mediated by other variables. In support of its proposed role in the aetiology of ED, alexithymia was directly associated with ED symptoms in women. It also affected ED symptoms indirectly, in all participants, via its effect on depressive/anxious symptoms. Interventions focusing on alexithymia may facilitate recovery not only via their effect on ED, but via their effect on other forms of state psychopathology which contribute to the maintenance and development of ED. Sex differences, however, reflect that alternative therapeutic targets for men and women may be beneficial.

**Supplementary Information:**

The online version contains supplementary material available at 10.1186/s40337-023-00746-7.

## Plain English Summary

Autistic individuals seem to be at higher risk of developing eating disorders (ED)—even just having autistic traits seems to elevate risk of ED, although we do not understand why. One possibility is that autism and autistic traits are closely related to alexithymia, a difficulty identifying and describing your emotions, and it may be this that increases risk of ED. To test this, we explored relationships between autistic traits and ED symptoms in men and women with ED, who completed an online survey. In men, we found that autistic traits were associated with ED symptoms *because* they were associated with alexithymia, and alexithymia was associated with ED symptoms *because* it was associated with anxious/depressive symptoms. The same was true in women, but anxious/depressive symptoms were also associated with ED symptoms in their own right. While these findings need to be investigated in more thorough longitudinal approaches, they suggest that pathways to ED differ slightly between men and women, autistic and non-autistic, and that therapeutic interventions should also differ. In both sexes, the fact that alexithymia was associated with ED symptoms *and* those of other mental illnesses that seemed to contribute to ED, suggests that it should be targeted in interventions.

**Supplementary Information:**

The online version contains supplementary material available at 10.1186/s40337-023-00746-7.

## Background

Eating disorders (ED) occur at higher rates in the autistic community and in those with greater autistic traits [1–5]. Moreover, such individuals often present with more complex, protracted symptoms and respond less favourably to some standard psychological interventions [6–8]. Efforts to understand the factors that predispose, precipitate and perpetuate ED in relation to autism are crucial in developing more effective and individualised clinical interventions and ultimately aiding treatment outcomes for this subgroup.

One thus far “understated” [9] factor in the development of disordered eating lies in atypical emotion processing and regulation. Alexithymia describes a cognitive style characterised by difficulties identifying and communicating one’s emotions and a tendency towards externally-orientated thinking [10]. People with high degrees of alexithymia are theorised to exhibit maladaptive tendencies throughout all stages of emotion processing [11], and consequently suffer higher stress exposure. Having reduced access to adaptive emotion-regulation strategies, they are more likely to rely on maladaptive and damaging strategies such as substance misuse, disordered eating and self-injury. Unsurprisingly, alexithymia (and corresponding emotion-regulation deficits) has been implicated in the aetiology of many psychopathologies and physical illnesses [10], including ED [12–14]. Alexithymia is also a common feature in autistic people, where it has been linked with a number of deleterious outcomes [15]—including disordered eating.

Recent literature suggests that alexithymia, and the maladaptive emotion regulation which appears to occur as a consequence of the same [13, 14], may comprise an emotion-related mechanism that explains the higher risk of disordered eating and ED in autistic people and those with higher autistic traits [16–19]. Indeed, in this latter population, previous data collected by our group [16] showed that the relationship between having higher autistic traits and higher eating psychopathology was explained by the higher degrees of alexithymia. Other studies similarly indicate a relationship between autistic features and eating psychopathology which is explained (mediated) by alexithymia or emotion dysregulation [17, 18].

These findings are potentially of broader clinical import for autistic *and* non-autistic people: they suggest that the risk of ED associated with autism and autistic traits might, in fact, be explained by the fact that autistic people and those with autistic traits are more likely to experience alexithymia. As such, emotion processing deficits could be a valuable target for intervention when occurring in autistic or non-autistic people alike. However, there are several limitations and unanswered questions which hinder interpretation of these findings. Firstly, all involved non-clinical undergraduate samples: while autism appears to exist on the same continuum as autistic traits [20], findings cannot necessarily be generalised to autistic people and/or people with formally-diagnosed ED. Secondly, men were under-represented in all three studies. While there is a lack of research around sex-specific aetiological factors for ED in the general population, clinical perspectives suggest that these are likely [21]; it is not presently clear if alexithymia and emotion difficulties contribute to male ED to the same extent [22]. This is indeed suggested in Vuillier et al. [16], who found no significant relationship between autistic traits and eating psychopathology (and no mediating effect via alexithymia) in their, albeit underpowered, male sample. Sex-moderated or sex-specific vulnerability factors for ED are also indicated in autistic people. In children, autistic girls^1^ were more likely than boys to over-eat as a means of emotion regulation [23]. Studying sex differences in eating behaviours and psychopathology in autistic children is complicated by the fact that autistic girls are likely to be diagnosed markedly later than autistic boys, especially if they have higher verbal and cognitive abilities [24–29]. In adolescence and adulthood, however, there is suggestion that autistic girls and women have more difficulty eating in social contexts than do boys and men [30]. As pertains to males, though, one recent study (albeit unpublished and not peer-reviewed) suggested a particular role for anxiety in relation to eating psychopathology in autistic men [31]. Unfortunately, in contrast to the previous studies, the authors did not include a female comparison group, or examine whether anxiety mediated relationships between autistic traits and eating psychopathology in a sex-specific way.

The third caveat of studies suggesting an emotion-related mediator of the relationship between autistic traits and ED psychopathology [16–18] is that they often failed to adequately separate out effects of alexithymia from those of anxiety and depression, which can inflate the relationship between ED symptomatology and autistic traits [32] or even give rise to the appearance of alexithymia in people with ED [33]. The latter view is contraindicated by studies showing the independence and perseveration of alexithymia in people recovered from ED [16, 34, 35], and by the dominant view of alexithymia as a personality trait which maintains a relative stability throughout the life-course and itself increases risk for depression and anxiety, alongside ED [10, 36]. From the standpoint that autistic traits and alexithymia are both relatively stable and closely related [37], it is possible that risk of ED associated with either anxiety or depression might, in fact, involve or be dependent on alexithymia as an additional, preceding mediator, but this has not been investigated.

The present study set out to test this precise hypothesis and remedy the aforementioned three limitations of previous studies in this field [16–18]. Firstly, we employed a clinical sample of individuals with eating disorders, and secondly aimed to re-examine previous hints of sex-specificity [16] with a larger male sample. Thirdly, following previous recommendations [18], the present study modelled other psychopathological states alongside alexithymia in order to better understand their role as potential mediators of the relationship between autistic traits and eating psychopathology. This approach aimed to establish whether effects of alexithymia on this central relationship were indeed independent of anxious-depressive symptoms (as per our previous study [16]). Although unable to measure temporal relationships between these variables, we also hoped to partly contribute to the debate around the role of alexithymia in the generation of psychopathological states besides ED (which might, indeed, themselves contribute to ED). We did this by modelling relationships between autistic traits and eating psychopathology via alexithymia, via anxious and depressive symptoms *and* via the two sequentially. While our analysis of males was not led by particular hypotheses due to the lack of work in this area, we hypothesised that in women, as in our previous study [16], effects of autistic traits on ED psychopathology would be mediated via alexithymia, as would effects of anxiety and depression on ED psychopathology (i.e. a sequential mediation).

## Methods

### Participants

The majority of participants (n = 456) were a convenience sample recruited for a study set in the UK during the COVID-19 pandemic [38]: they were originally recruited from a participant recruitment website (Prolific) and through social media. The remaining participants (n = 22) were recruited from the undergraduate cohort at Bournemouth University. As this previous work indicated that many people suffering from an ED were unable to access health services and diagnostic assessments during the pandemic [38], and since it was unfeasible for the present authors to interview all participants to establish diagnosis, the present investigation includes both participants who reported that they had been formally diagnosed with an ED by a clinician, and those who had never received a formal ED diagnosis but suspected that they might now be suffering from an ED which had not (yet) been diagnosed. In order to have large, well-matched groups, we ensured all participants were cisgender men and women.

After removing incomplete datasets, the total sample comprised 198 cisgender male participants (108 formally diagnosed, 90 ED-suspecting) and 265 cisgender female participants (155 formally diagnosed, 110 ED-suspecting), whose ages were 31.09 years (SD: 9.9) and 29.48 years (SD: 9.6) respectively (no significant difference [p = 0.09]). Most participants were white British or white European (88.34%), with others describing themselves as Black British (0.86%), Asian British (2.37%), Asian (3.01%), Arab (0.65%), Chinese (0.65%), Black African (0.43%), Greek (0.22%), Latino (0.22), mixed race (2.15%), or choosing not to specify. All were qualified to GCSE level (UK secondary education) or equivalent, with many male (45.22%) and female participants (48.12%) studying for or qualified to degree level. Just over four percent of the total male sample and 4.14% of the female sample self-reported that they had received a formal diagnosis of autism in the past.

We were unable to validate diagnoses within this study, but participants who reported that they had been formally diagnosed with an ED told us their bestowed diagnoses as follows: anorexia nervosa (30.28% of males, 39.74% of females), bulimia nervosa (26.61% of males, 20.51% of females), binge-eating disorder (26.61% of males, 15.38% of females), OSFED or EDNOS (3.67% of males, 10.90% of females), orthorexia nervosa (0.92% of males, 1.28% of females). The remainder (11% of males, 11.55% of females) expressed that they had had multiple diagnoses within the ED spectrum (e.g. anorexia nervosa and bulimia nervosa at different points) or failed to clearly specify their diagnosis.

### Measures and procedure

Approved by the Faculty of Science and Technology Ethics Panel at the first author’s institution, the study occurred between 05/2020 and 07/2021. Participants completed a number of standardised questionnaires hosted on an online platform, Qualtrics, which included the following constructs as predictors, mediators and dependent variables:

#### The Autism-Spectrum Quotient, short form (AQ-S)

The abridged AQ-short (AQ-S) [39] boasts good sensitivity, specificity and internal consistency (α at 0.79 and 0.83 for males and females in the present study). Its 28 items are scored on a 4 point Likert scale, with higher scores (> 65) indicative of higher degrees of autistic traits.

#### The Toronto Alexithymia Scale (TAS-20)

The TAS-20 [40] is the most commonly used measure of alexithymia [41]. Its total score of all 20 items boasts strong internal consistency (α = 0.82 and 0.86 for our male and female samples), with higher scores indicative of greater degrees of alexithymia (scores > 65 indicative of clinically-significant alexithymia).

#### The Depression, Anxiety and Stress Scale (DASS-21)

The DASS-21 comprises subscales measuring symptoms of depression, anxiety and stress experienced over the past week. Higher scores in each subscale are indicative of greater symptoms in that domain, with a suggested cut-off of > 16 (total score) indicative of anxiety disorders or depression [42]. Scores for each subscale derive from the sum of items (7 per subscale) multiplied by two (as the DASS-21 is a short form of the original scale, which is precisely twice as long [43]). The DASS-Total score similarly comprises the sum of all 21 items multiplied by 2.

Recently, concerns have been raised regarding the three-factor structure of the DASS-21 [44, 45]: data across multiple studies is more suggestive of a bifactor model comprising a *general* factor (reflecting “a mixture of negative emotions such as depression, anxiety, and [being] stress-reactive” (45, p. 166)) and a *group* factor (including all three subscales or just the two most independent: depression and anxiety). Due to these psychometric concerns, the present analysis utilised DASS-Total scores as a reflection of these psychopathological states, albeit a non-specific one as regards to differentiating between anxious and depressive symptoms. It boasted good internal consistency in both the male (α = 0.91) and female (α = 0.93) samples. Secondary analyses were performed to examine these two subscales independently (see Analysis and Additional file 1).

#### The Eating Disorder Examination Questionnaire (EDEQ)

The EDEQ [46] comprises 28 items describing ED psychopathology over the past month. Used as our dependent variable, the total score demonstrated strong internal consistency in our sample (α = 0.93 in males, 0.92 in females). The traditional cut-off score (> 4) indicative of a clinically significant ED was met by 71 of our male group (28 ED-suspecting, 43 formally diagnosed) and 158 of our female group (60 ED-suspecting, 98 formally-diagnosed), but some suggest this threshold is ill-suited for global application across all types of ED in people of different sizes, ages and ethnicities [47]. Males, in particular, may require cut-offs as low as 1.68 [48], or 2.41 [49]. There was no significant difference in total EDEQ score between formally diagnosed and ED-suspecting female participants (F [1, 264] = 3.22, p = 0.074).^2^ Our male diagnosed participants were experiencing significantly greater ED psychopathology than males with suspected ED (F [1, 197] = 6.66, p = 0.011), but the average scores of both were similar to those in reported literature [50] and above the newly-suggested cut-offs [48, 49], indicating that both male groups were experiencing marked ED psychopathology.

### Analysis

**Fig. 1:**
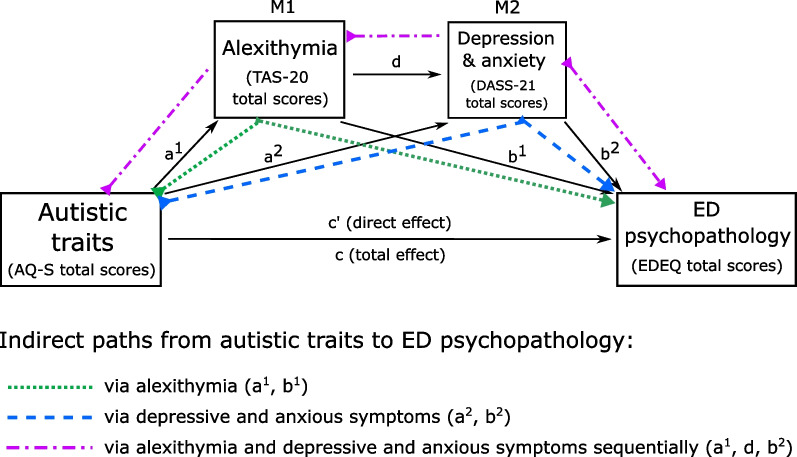
Conceptual model of sequential mediation. Conceptual diagram of sequential mediation model as computed with PROCESS Model 6

We visually inspected the data for linearity between predictors, mediators and EDEQ scores, and for normality of residuals and homoscedasticity. The data was also screened for outliers (Cook’s test), autocorrelation (Durbin-Watson test), and for multicollinearity between the three predictor variables (AQ-S scores, TAS-20 scores, and DASS-Total scores) in the male and female groups (VIF values < 1.5). Analysis was performed using the PROCESS macro for SPSS [51], which is based on ordinary-least-squares regression with bootstrapping (here set at 5000 samples). Model 6 in PROCESS reveals direct effects of autistic traits on ED psychopathology (with the influence of mediators controlled for), if present. However, and in accordance with the hypothesised causal role of alexithymia in psychopathological states [10, 11, 41], it also allowed us to examine indirect effects of autistic traits on ED psychopathology a) via alexithymia (M1: TAS-20 scores) independently, b) via anxious-depressive symptoms (M2: DASS-Total scores) independently, or c) via alexithymia and through that, depressive and anxious symptoms (a two-step sequential mediation pathway through M1 and M2; see Fig. 1). As such, the analysis was able to indicate not only whether alexithymia was a necessary mediator in bridging the relationship between autistic traits and eating psychopathology, but whether additional mediation effects of anxious-depressive symptoms were independent of alexithymia or themselves dependent on alexithymia sequentially. The analysis also yielded a total effect of autistic traits on ED psychopathology, which reflects both direct *and* indirect effects (i.e. through mediator variables) of autistic traits on ED psychopathology. We include this for completeness, though in accordance with the Hayes approach [51], the occurrence of mediation is indicated by confidence intervals for each indirect pathway.

This analysis was performed once for female and once for male participants (alpha levels corrected to p = 0.025, with confidence intervals set to 95%). Analyses were then corroborated using only those participants with formal ED diagnoses (see Additional file 1: 1), and replacing DASS-Total scores with those from the DASS-Anxiety and DASS-Depression subscales (see Additional file 1: 2).

## Results

The scores of each group were calculated for each major study variable and are presented in Table 1. Higher than average population levels of autistic traits, anxious-depressive symptoms and alexithymia were evident across all participant groups, all of whom had marked levels of ED psychopathology in accordance with recommended sex-adjusted cut-offs [48, 49].

**Table 1:** Sample demographics and scores in experimental variables

	Male sample			Female sample		
	Diagnosed (n = 108)	Suspected ED (n = 90)	Total (n = 198)	Diagnosed (n = 155)	Suspected ED (n = 110)	Total (n = 265)
Age	30.11 (9.4), *18–55*	32.26 (10.36), *18–58*	31.09 (9.9), *18–58*	29.97 (9.3), *2–59*	28.79 (10.15), *18–69*	29.48 (9.6), *18–69*
AQ-Short total	74.03 (11.3), *46–107*	71.98 (11.22), *46–100*	73.10 (11.3), *46–107*	70.06 (13.4), *38–106*	67.98 (10.76), *43–95*	69.20 (12.4), *38–106*
Percentage over cut-off (> 65)	78.81	73.33	78.88	58.97	60	59.40
TAS-20 total	64.28 (11.1), *29–85*	60.31 (12.51), *28–84*	62.47 (11.9), *28–85*	61.34 (12.5), *28–88*	58.76 (12.41), *34–83*	60.27 (12.5), *28–88*
Percentage over cut-off (> 61)	63.30	51.11	57.78	50.64	43.64	47.74
EDEQ total	3.75 (1.1), *1.32*	3.29 (1.32), *.27–6*	3.54 (1.21), *.27–6*	4.19 (1.1), *.68–6*	3.93 (1.15), *.73–5.8*	4.08 (1.1), *.68–6*
DASS total score	68.67 (24.1), *6–120*	64.27 (22.06), *14–124*	66.67 (23.3), *6–124*	66.00 (24.0), *14–120*	61.51 (25.67), *10–126*	64.1 (24.7), *10–126*
Percentage over cut-off (> 16)	99.08	98.88	98.99	98.08	94.55	96.62

Average scores in each variable are shown with standard deviations in brackets and range in italics

**Fig. 2:**
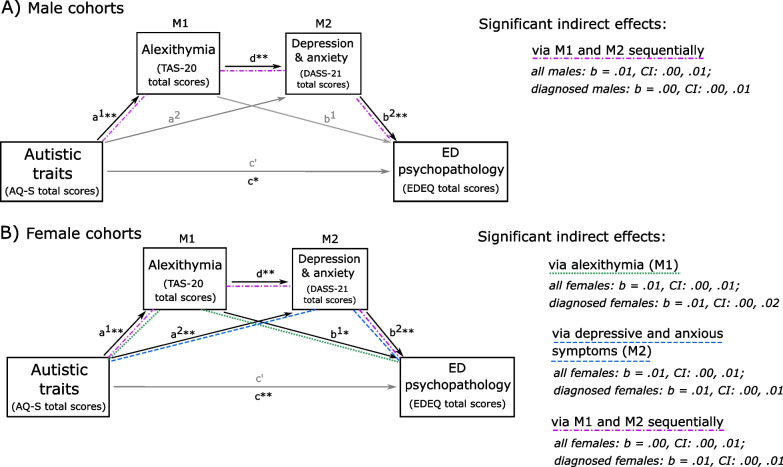
Indirect effects of autistic traits on ED psychopathology. A depicts associations between autistic traits, alexithymia, depressive and anxious symptoms (DASS-Total scores), and eating psychopathology in the male groups (entire and formally-diagnosed only); B Depicts the same relationships in the female groups (entire and formally-diagnosed only). In each instance, double asterisks reflect relationships that were significant at our corrected alpha level (p < .025); a single asterisk marks out relationships significant at the traditional p < .05

For males, the sequential mediation model for the total cohort showed a significant relationship between autistic traits and levels of alexithymia (path a^1^: *b* = 0.38, p < 0.001; *R*^*2*^ = 0.13, *F* [1, 196] = 29.49, *p* < 0.001). However, no significant correlation was found between autistic traits and DASS-Total scores (path a^2^: *b* = 0.23,* p* = 0.0847), which were instead wholly predicted by alexithymia (path d: *b* = 0.97, *p* < 0.001; *R*^*2*^ = 0.30,* F* [2, 195] = 41.97, *p* < 0.001). In the model predicting ED psychopathology (*R*^*2*^ = 0.17, *F* [3, 194] = 12.96,* p* < 0.001), only higher DASS-Total scores significantly predicted greater eating psychopathology (path b^2^:* b* = 0.02, *p* < 0.001); neither the direct effect of autistic traits (path c’: *b* = 0.00, *p* = 0.7475) nor alexithymia (path b^1^: *b* = 0.01, *p* = 0.1885) significantly contributed to the model. The total effect of autistic traits on disordered eating was just over our corrected significance level when both alexithymia and DASS-Total were uncontrolled for as mediators (path c: *b* = 0.02, *p* = 0.0262, *R*^*2*^ = 0.02, *F* [1, 196] = 1.60, *p* = 0.0262). Of the three potential indirect effects that were hypothesised, only the two-step indirect pathway was significant, indicating that autistic traits were associated with greater ED psychopathology through higher levels of alexithymia and through that higher DASS-Total scores sequentially (*b* = 0.01, CI: 0.00, 0.01). These findings were near exactly replicated in the formally-diagnosed male sample (see Additional file 1: 1), with the same sequential indirect effect occurring. Relationships between variables are displayed in Fig. 2, with 2A reflecting findings in male participants and 2B reflecting those in females.

For the female sample, as in males, analysis of the combined cohort revealed significant associations between autistic traits and alexithymia (path a^1^: *b* = 0.48, *p* < 0.001; *R*^*2*^ = 0.23,* F* [1, 263] = 77.92, *p* < 0.001). In contrast to male participants, however, autistic traits were also associated with DASS-Total scores (path a^2^: *b* = 0.40, *p* = 0.0010; *R*^*2*^ = 0.27, *F* [2, 262] = 48.46, *p* < 0.001), which were also contributed to by alexithymia (path d: *b* = 0.78, *p* < 0.001). While as per the male group there was no significant direct effect of autistic traits on ED psychopathology (path c’:* b* = 0.00, *p* = 0.8323; *R*^*2*^ = 0.15, *F* [3, 261] = 15.43, *p* < 0.001), both DASS-Total scores (path b^2^: *b* = 0.01, *p* < 0.001) and TAS-20 scores (path b^1^: *b* = 0.01, *p* = 0.0332) contributed to the model, although the latter had no significant effect after statistical correction to* p* < 0.025. A significant total effect of autistic traits on ED psychopathology (path c:* b* = 0.02, *p* = 0.0013; *R*^*2*^ = 0.04,* F* [1, 263] = 10.50, *p* = 0.0013) was underpinned by three indirect effects (Fig. 2, Part B): that is, autistic traits exerted their effects via alexithymia alone (*b* = 0.01, CI: 0.00, 0.01), via DASS-Total scores alone (*b* = 0.01, CI: 0.00, 0.01), and via alexithymia and then DASS-Total scores sequentially (*b* = 0.00, CI: 0.00, 0.01). Relationships between variables and all three indirect effects remained significant in formally-diagnosed females (see Additional file 1).

## Discussion

The present study re-examined relationships between autistic traits and ED symptoms in a clinical sample, one more balanced in its male/female ratio, and paying particular attention to mediation by and interactions between alexithymia and depressive and anxious symptoms. With respect to the first of these aims, our analysis confirmed the existence of an association, a total effect, of autistic traits on ED psychopathology in a sample with clinically substantive ED [52]. To increase statistical power, we collated the formally diagnosed and ED-suspecting groups (whose group average EDEQ scores were in fact comparably high). The consistency across our analyses supported this decision. While total effects are suggestive of an association between autistic traits and ED psychopathology ‘on the surface’, closer inspection of indirect and direct effects revealed that in each group, the effect of autistic traits on ED psychopathology was indirect, dependent on the associations of these variables with our mediators.

Our second goal had been to examine these relationships in cisgender men as well as cisgender women, since the former are under-represented in ED research. Our data corroborated the few existing studies purporting an association between autistic traits (or diagnosed autism) and ED psychopathology in men [30, 53, 54], but again showed that this relationship was only an indirect one, carried by intermediary variables. In contrast to the smaller, non-clinical male group of Vuillier et al. [16], in this male sample autistic traits *were* associated with ED psychopathology (at least ostensibly, in the total effect, though not in fact when the direct effect was examined). So too was alexithymia, although the effect of alexithymia was only indirect via its effect on anxious/depressive symptoms. In female participants, while autistic traits were not directly associated with ED psychopathology, they were indirectly associated with this outcome variable via alexithymia, via depressive and anxious symptoms independently, and also via the sequential effect of *alexithymia* on depressive and anxious symptoms. The differences between groups are indicative of sex-specificity in the intermediary variables which link autistic traits and ED psychopathology. These findings are supportive of the independence of alexithymia from contributions of depression and anxiety, something which was not fully clear from our previous work [16]; however, they also indicate that these other variables, in women at least, may mediate the association between autistic traits and ED psychopathology in their own right. To fully review this finding in relation to our third goal, we must revisit the literature concerning our focal mediators, their relationships with one another, and their potential roles in the risk of ED psychopathology.

## Alexithymia, anxiety and depression; their relation to ED psychopathology and autistic traits

Conceptually, mediators are of interest as the causal links or mechanisms through which a certain feature (in this instance, autism or autistic traits) increases vulnerability for certain psychopathologies (in this case, ED). Both alexithymia [37, 55] and anxious/depressive symptoms are common in autistic people and individuals with high degrees of autistic traits [56], and in populations with ED [13, 33, 34]. Like non-autistic counterparts [57], autistic participants with anorexia nervosa describe feelings of ‘emotional confusion’ and anxiety which are quietened or numbed through restrictive behaviours [58]. Elsewhere, autistic adults have likewise recognised a role for anxiety and depression in “emotional eating” and binge-eating [59]. Problems with emotion identification and regulation, along with anxiety and vulnerability to negative emotions coupled with a drive for emotional avoidance, have been accorded a central role in the aetiology and maintenance of ED [60]. If alexithymia, anxiety and depression are risk factors for ED and tend to be more prevalent in autistic people and those with higher autistic traits, then, firstly, it follows that the risk of ED would also be higher in those groups. Secondly, it follows that this risk may occur via the effects of alexithymia and/or symptoms of anxiety or depression, rather than being associated with autism or autistic traits in and of themselves.

The dovetailing of these mediators is inherent in the cognitive-interpersonal approach described above [60] and implied in the lived-experience perspectives of autistic and non-autistic people with ED [57, 58]. However, causal primacy between these mediators is conceptually debated: some suggest alexithymia is a relatively stable pre-morbid trait which engenders anxiety, depression, and other psychopathological states [10, 11, 13, 41], while others suggest that alexithymia may be a consequence or reflection of state psychopathology, including ED [33]. While longitudinal research is needed to understand causal relationships between these factors and their role in the aetiology of ED, we hoped to contribute to this debate by modelling contributions from depressive and anxious symptoms as mediators in their own right. This would not only confirm whether effects of alexithymia were indeed independent from depressive-anxious symptoms, but would also show whether these forms of state psychopathology are themselves a necessary step (i.e. a mediator) in the association between autistic traits and ED psychopathology, *or* whether they only affect ED symptomatology through their association with alexithymia.

While our analysis suggests that alexithymia may influence ED in its own right, these indirect pathways differed between female and male participants, consistent with sex-specificity in aetiological factors for ED [16, 21]. The presence of *three* indirect pathways from autistic traits to ED psychopathology, in females, supports the importance of both alexithymia and of anxious-depressive symptoms as mediators of this relationship. The two-step sequential pathway supports the theorised role of alexithymia in the generation of psychopathological states other than ED [11], but anxious and depressive symptomatology is unlikely to be wholly explained by alexithymia in each and every case. The independent mediating effect of DASS-Total scores in our female participants could reflect that autistic traits are associated with a wider range of factors associated with negative affect, including social anxiety and low self-worth [61], which could drive an individual towards emotion-regulating ED behaviours. Interestingly, and in contrast to the female cohorts, in male participants autistic traits were related to symptoms of anxiety and depression (and ED psychopathology) *only* via alexithymia. It is possible that forms of negative emotionality less strongly associated with alexithymia play a smaller role in male ED, but this suggestion is highly tentative. However, our findings indicate that the association between ED psychopathology and anxiety formerly seen in autistic men [31] might be usefully re-examined with reference to alexithymia [62].

In summary, our data is supportive of the importance of alexithymia in ED [13, 60], although its mediating role in the autism-ED relationship, in female participants at least, may be parallel to independent contributions from depressive and anxious symptomatology. This contribution from alexithymia would explain why, in previous samples of men and women from the general population, an association between autistic traits and ED psychopathology [53] (and that we observed indirectly via alexithymia [16]) survived once controlling for anxiety and depression as potential confounds. In relation to ED and broader psychopathology, our findings support previous suggestions [10, 15, 36, 37] that alexithymia is an important factor to, at the very least, accommodate in psychological interventions for ED [63], and which could furthermore be a valuable therapeutic target in autistic and non-autistic people alike.

### Limitations and future directions

While cross-sectional designs are an effective way of testing hypotheses in large datasets, they cannot establish causal primacy or the dispositional stability of autistic traits or alexithymia [20, 41]. Nor could we establish that autistic traits or alexithymia, in these samples, were premorbid rather than the effect of malnutrition. It is necessary, as such, to re-examine whether these findings can be reproduced in more controlled longitudinal settings, ideally with a comparison sample. Future studies should also independently validate the presence and nature of ED diagnoses using structured standardised clinical interviews.

The generalisability of our sample is limited with regards ethnicity (participants being mainly British and Caucasian), and gender. In that autistic people are more likely to be gender-variant [65], our exclusion of gender minorities is especially important to rectify in future research. As the factors which mediate the risk of ED psychopathology may differ in autistic people vs. those with varying levels of autistic traits, these findings should be validated in autistic cohorts, ideally in designs which reflect the developmental trajectory of ED psychopathology. While we focused here on emotion-related mechanisms, a number of other factors have been recognised as risk factors for the development of ED in autistic people [1, 9, 52, 58], and so should be considered in future investigations.

We adopted a well-supported transdiagnostic conceptualisation of ED [12, 66], which purports similar aetiological factors at play across the ED spectrum, but there is evidence of some differences in the profiles of emotion difficulties across different ED diagnoses [67]. There is also indication that autistic traits might be differentially associated with different features of ED symptomatology [2, 17]. Unfortunately, our study lacked the power to examine effects within each ED diagnostic category, and our combining participants may have obscured relationships between variables that might have emerged more clearly in relation to specific ED behaviours. While research in this area has focused on anorexia nervosa, recent studies recognise that other manifestations of ED may also occur at higher rates in autistic people and those with high autistic traits [31, 59]—these distressing conditions, and the factors that mitigate increased risk of the same, are an important focus for future research.

With regard to our psychometric measures, it is notable that alexithymia may also affect a person’s ability to judge their own emotional awareness, and there is a lack of consensus around optimal measurement of this construct in minority groups such as autistic people or those with high autistic traits [68]. This and other research [62] are beginning to indicate that emotion-related mechanisms are relevant for ED in males, too, but future research in this group should recognise possible impacts of cultural masculine norms of ‘emotional inexpressiveness’ or ‘restrictive emotionality’, which can affect responses to the TAS-20 [69]. With reference to the DASS-21, the present study followed previous trends of collapsing depressive and anxious symptoms into a single factor [18, 53], and so could not ascertain any differential effects of anxious and/or depressive symptoms. Furthermore, given that different scales are weighted towards somatic vs. cognitive-affective symptoms, they may interact differently with measures of autistic traits and alexithymia. This could partly explain why this study and others [16] report relationships between autistic traits, alexithymia and ED psychopathology that are independent from anxiety and depression, while others do not [18, 33].

## Conclusions

While the present findings require replication with more rigorous experimental designs capable of confirming directionality, they support previous indications that the relationship between autistic traits and ED psychopathology, apparent in our total effect and in other studies, is actually dependent on intermediary factors. In real terms, this means that autistic traits may be associated with ED psychopathology *because* they are likely to co-occur with alexithymia (which may have its own psychopathological sequelae), and with anxious/depressive symptoms. These findings are consistent with theoretical approaches purporting the importance of emotion awareness, and emotion regulation, in the psychopathology of ED; they indicate that the alexithymia and anxious/depressive symptomatology associated with autistic traits (and by extension, autism) may play an important role in heightened risk of ED. While this highlights the importance of these variables in psychological interventions, the sex differences we observed between key study variables corroborates the need for sex- and gender-informed treatment. For men, as alexithymia affected ED symptoms indirectly via its association with anxious/depressive symptomatology, interventions focusing on awareness, identification and expression of emotion may facilitate recovery not only via their effect on ED psychopathology, but via their effect on other psychopathological states which contribute to ED. In women, while alexithymia appeared to contribute to the psychopathological states that factor into ED symptoms (as well as directly affecting ED psychopathology), these anxious/depressive symptoms appeared to have broader origins which would necessitate intervention in order to ameliorate their effects on ED.

## Supplementary Information

**Additional file 1.** Supplementary analyses.

## Data Availability

The datasets used and/or analysed during the current study are available from the corresponding author on reasonable request.

## Abbreviations

AQ-S: Autism-Spectrum Quotient short-version (28 items)
ARFID: Avoidance/Restrictive Food Intake Disorder
ASC: Autism Spectrum Conditions
BMI: Body Mass Index
DASS-21: Depression, Anxiety and Stress Scales (21 item version)
ED: Eating disorders
EDEQ: Eating Disorder Examination Questionnaire
EDNOS: Eating Disorder Not Otherwise Specified, the term used in DSM-IV prior to ‘OSFED’
OSFED: Other Specified Feeding and Eating Disorders, the equivalent to ‘EDNOS’ as per DSM-5
TAS-20: Toronto Alexithymia Scale (20 item version)

## Footnotes

1 Gender dysphoria and non-binary gender identities are more common in autism and may be associated with unique challenges and mental health difficulties (George and Stokes, 2017). Though with ‘women’ and ‘girls’ we use the terminology employed by Wallace et al. and by Remnelius et al., it is important to note that these studies assumed gender identity in participants.

2 Formally diagnosed and ED-suspecting participants were also compared with respect to other major variables in the study. Between women who were formally diagnosed and those who were ED-suspecting, there were no significant differences in AQ-S, DASS-21 or TAS-20 scores. In the male group, formally diagnosed men scored significantly higher than ED-suspecting men on the TAS-20 (F [1, 198] = 5.131, p = .025), but did not differ significantly in DASS-21 or AQ-S scores. There were no significant age differences between formally-diagnosed and ED-suspecting samples.
