# Supplementary material for: Sex-specific mechanisms for eating disorder risk in men and women with autistic traits: the role of alexithymia
Source: J Eat Disord. 2023 Feb 10;11:18. doi: 10.1186/s40337-023-00746-7 (PMC9912205; doi:10.1186/s40337-023-00746-7)
Supplement: Supplementary file 1 — Additional file 1. Supplementary analyses. [file 40337_2023_746_MOESM1_ESM.docx]

**Supplementary materials**

1. Analyses with formally diagnosed participants only
2. Analyses with DASS-Anxiety and DASS-Depression as mediators
3. **Analyses with formally diagnosed participants**

Our principle analyses, which modelled the sequential mediation effects of autistic traits via alexithymia and then DASS-Total scores (PROCESS Model 6), were as follows for formally-diagnosed groups:

*Male participants*

The pattern of relationships between variables, in formally-diagnosed males, was almost wholly identical to that seen in the larger cohort comprising both formally-diagnosed and ED-suspecting males. Just as in the principle analysis, autistic traits were associated with alexithymia, the first mediator (path a^1^: *b =* .24, *p* = .0104; *R^2^* = .06, *F* [1, 106] = 6.81, *p* = .0104), but not quite significantly associated with DASS-Total scores (path a^2^: *b* = .35, *p* = .0583), which were predicted solely by alexithymia (path d: *b* = 1.05, *p* < .001; *R^2^* = .30, *F* [2, 105] = 22.22, *p* < .001). As in the combined analysis, the model for ED psychopathology was contributed to solely by DASS-Total scores (path b^2^: *b* = .02, *p* = .0005; *R^2^* = .17, *F* [3, 104] = 6.99, *p* = .0002). While in the entire male sample the total effect (c) had been just over our corrected alpha level (*p* = .0262), the total effect of autistic traits on ED psychopathology was in this instance non-significant in the smaller diagnosed group (path c: *b* = .02, *p* = .0939; *R^2^* = .03, *F* [1, 106] = 2.86, *p* = .0939). However, the observed two-step mediation effect of autistic traits, via alexithymia and through that DASS-Total scores, was still evident (*b* = .00, CI: .00, .01), such that mediation was interpreted to have occurred. While in some approaches mediation is contingent on the significance of the total effect in contrast to the direct effect (e.g. the causal steps approach), other perspectives, including that underpinning PROCESS that we employed, consider mediation to have occurred based on the existence of indirect effects, since total effects can drop below significance or be suppressed for a number of reasons (Rucker et al., 2011).

*Female participants*

Relationships between variables were precisely preserved in the smaller formally-diagnosed female cohort. Autistic traits predicted alexithymia (path a^1^: *b* = .41, *p* < .001; *R^2^* = .20, *F* [1, 153] = 37.45, *p* < .001) and DASS-Total scores (path a^2^: *b* = .42, *p* = .0010), which were also predicted by alexithymia (path d: *b* = .90, *p* < .001; *R^2^* = .38, *F* [2, 152] = 47.15, *p* < .001). The model for ED psychopathology (*R^2^* = .17, *F* [3, 151] = 10.43, *p* < .001) was contributed to by DASS-Total scores (path b^2^: *b* = .01, *p* = .0023) and by alexithymia (path b^1^: *b* = .02, *p* = .0311), though this relationship did not survive our corrected threshold (*p* = .025). The one difference between the combined and the formally-diagnosed group lay in the total effect (c) of autistic traits on ED psychopathology, which was non-significant in the latter (*b* = .01, *p* = .0544; *R^2^* = .02, *F* [1, 153] = 3.76, *p* = .0544), but the three indirect effects exerted by autistic traits on ED psychopathology remained significant: via alexithymia alone (*b* = .01, CI: .00, .02), via DASS-Total scores (*b* = .01, CI: .00, .01), and via alexithymia and then DASS-Total sequentially (*b* = .01, CI: .00, .01). Mediation was thus interpreted to have occurred.

1. **Analyses with DASS-Anxiety and DASS-Depression as mediators**

Scores to the DASS-Anxiety and DASS-Depression subscales showed high internal consistency in our male (α = .83 for DASS-Anxiety, .86 for DASS-Depression) and female samples (α = .85 for DASS-Anxiety, .89 for DASS-Depression). Descriptive statistics for these subscales were as follows:

|  | **Male sample** | | | **Female sample** | | |
| --- | --- | --- | --- | --- | --- | --- |
|  | Diagnosed  (n = 108) | Suspected ED  (n = 90) | Total  (n = 198) | Diagnosed  (n = 155) | Suspected ED  (n = 110) | Total  (n = 265) |
| DASS Anxiety | 19.61 (9.22), *0-42* | 15.49 (9.34), *0-42* | 17.75 (9.47), *0-42* | 17.24 (9.38), *2-40* | 16.55 (10.40), *0-42* | 16.95 (9.80), *0-42* |
| DASS Depression | 25.17 (8.13), *4-42* | 25.62 (9.26), *2-42* | 25.38 (8.64), *2-42* | 24.22 (9.83), *2-42* | 22.53 (9.71), *2-42* | 23.52 (9.80), *2-42* |

The sequential mediation models of our principle analysis were repeated first with DASS-Anxiety scores as the second sequential mediator (PROCESS Model 6), and then with DASS-Depression as the second sequential mediator. While associations between autistic traits and alexithymia remained consistent as presented in the primary analysis and Supplementary 1, associations between autistic traits and the second mediator, and between this second mediator and ED psychopathology, are presented comparatively below.

**Male cohorts**

*DASS-Anxiety*

Regardless of whether DASS-Anxiety or DASS-21 scores were used as the second mediator, the sequential mediation model for the male cohorts was highly similar (see Figure 3, Part A). As in our main analysis, autistic traits were not significantly associated with anxiety (path a^2^) in the whole male cohort (*b* = .04, *p* = .7070; *R^2^* = .27, *F* [2, 195] = 35.33, *p* < .001) OR the formally-diagnosed group (*b* = .11, *p* = .1365). In both cohorts, DASS-Anxiety was predicted only by alexithymia (path d, whole group: *b* = .40, *p* < .001; diagnosed group: *b* = .37, *p* < .001). In both the entire male group and the formally-diagnosed male group, and just as seen in the DASS-21 analysis, the model predicting ED psychopathology was contributed to only by anxiety, though this fell below significance in the diagnosed group (whole group: *b* = .03, *p* = .0012; *R^2^* = .14, *F* [3, 194] = 10.31, *p* < .001; diagnosed group: *b* = .03, *p* = .0321; *R^2^* = .10, *F* [3, 103] = 4.13, *p* = .0085).

Figure 3


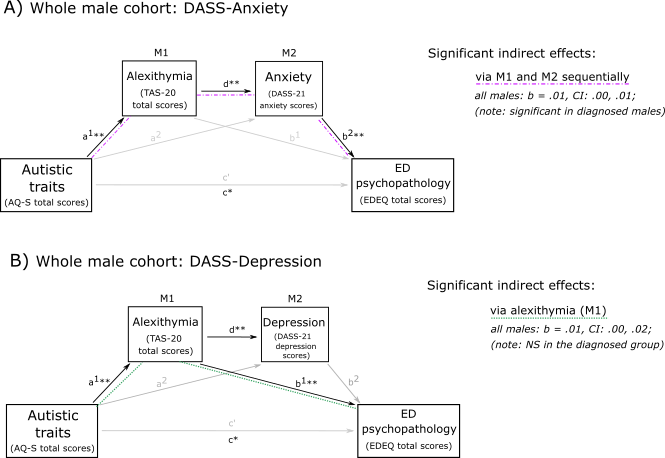


Figure 3: Sequential mediation with a) DASS-Anxiety and b) DASS-Depression in the whole male cohort. Significant relationships (p < .025) are marked with two asterisks; relationships significant in accordance with traditional alpha levels (p < .05) are marked by a single asterisk. Note that although only the whole group are shown, for simplicity, there were some minor differences in the formally-diagnosed group; we have indicated where indirect effects were still significant.

For the whole male group, the total effect of autistic traits of ED psychopathology (path c: *b* = .02, *p* = . 0262; *R^2^* = .02, *F* [1, 196] = 5.02, *p* = .0262) was, as in the main analysis, mediated only by the two-step indirect effect of autistic traits via alexithymia and then anxiety (*b* = .01, CI: .00, .01). Just as in the previous analysis with the diagnosed group, the total effect (c) of autistic traits on ED psychopathology fell below significance (*b* = .02, *p* = .0939; *R^2^* = .03, *F* [1, 106] = 2.86, *p* = .0939), but that same two-step indirect effect, via alexithymia and then DASS-Anxiety sequentially, remained significant (*b* = .00, CI: .00, .01).

*DASS-Depression*

For males, DASS-Depression scores operated similarly to DASS-Anxiety and DASS-21 scores as regards their association with autistic traits (see Figure 3, part B). They were not significantly related to autistic traits (path a^2^) in the whole male cohort (*b* = .07, *p* = .2327) or the formally-diagnosed cohort (*b* = .07, *p* = .3313); in both instances, DASS-Depression scores were predicted only by alexithymia (path d, all males: *b* = .24, *p* < .001; *R^2^* = .14, *F* [2, 195] = 15.66, *p* < .001; diagnosed males: *b* = .25, *p* = .0004; *R^2^* = .14, *F* [2, 105] = 8.55, *p* = .0004).

While formerly DASS-21 and DASS-Anxiety scores had been the only significant (or near-significant) predictor in the model for ED psychopathology in both the whole male and diagnosed male groups, with DASS-Depression as the second mediator, only alexithymia significantly contributed to the model of ED psychopathology (path b^1^) in the whole male cohort (*b* = .02, *p* = .0022; *R^2^* = .10, *F* [3, 194] = 7.02, *p* = .0002); in the formally-diagnosed group, all of the individual predictors were non-significant despite a significant model (*R^2^* = .09, *F* [3, 104] = 3.35, *p* = .0219). While the total effect of autistic traits on ED psychopathology (path c: *b* = .02, *p* = .0262; *R^2^* = .02, *F* [1, 196] = 5.0175, *p* = .0262), in the entire male group, was underpinned by an indirect mediation effect via alexithymia alone (*b* = .01, CI: .00, .02), the total effect of autistic traits on ED psychopathology in the diagnosed group was non-significant (*b* = .02, *p* = .0939; *R^2^* = .03, *F* [1, 106] = 2.86, *p* = .0939), with no significant indirect effects.

**Female cohorts**

*DASS-Anxiety*

While autistic traits had been predictive of DASS-21 scores when modelled as the second mediator, they were not significantly predictive of DASS-Anxiety in the whole female group (path a^2^: *b* = .08, *p* = .1244) or the formally-diagnosed participants (*b* = .09, *p* = .0871); in both instances, alexithymia was the only significant predictor of DASS-Anxiety (path d, whole group: *b* = .28, *p* < .001; *R^2^* = .17, *F* [2, 262] = 27.44, *p* < .001; diagnosed group: *b* = .31, *p* < .001; *R^2^* = .25, *F* [2, 152] = 24.82, *p* < .001). (See Figure 4).

Figure 4


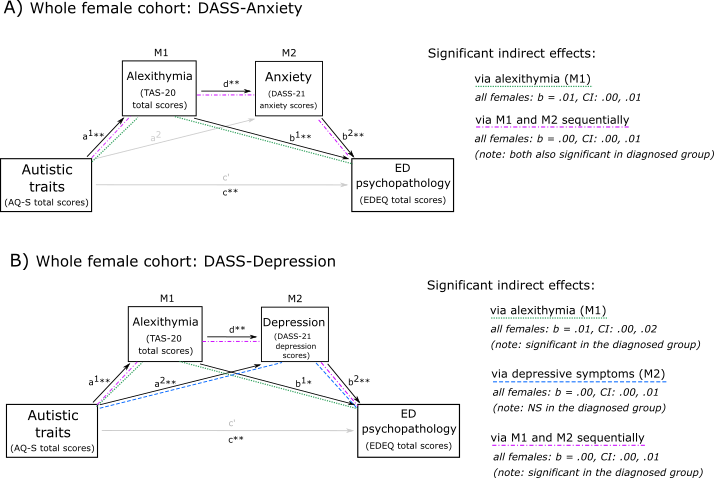


Figure 4: Sequential mediation with a) DASS-Anxiety and b) DASS-Depression in the whole female cohort. Significant relationships (p < .025) are marked with two asterisks; relationships significant in accordance with traditional alpha levels (p < .05) are marked by a single asterisk. Note that although only the whole group are shown, for simplicity, there were some minor differences in the formally-diagnosed group; we have indicated where indirect effects were still significant.

In both the entire female cohort and the formally-diagnosed group, the model for ED psychopathology (whole group: *R^2^* = .15, *F* [2, 261] = 14.81, *p* < .001; diagnosed group: *R^2^* = .18, *F* [3, 151] = 10.77, *p* < .001) was contributed to by both anxiety (path b^2^, whole group: *b* = .03; *p* = .0001; diagnosed group: *b* = .03, *p* = .0014) and alexithymia (path b^1^, whole group: *b* = .02, *p* = .0143; diagnosed group: *b* = .02, *p* = .0124). In both instances, there was no significant direct effect of autistic traits. In the entire female group, the total effect of autistic traits on disordered eating (path c: *b* = .02, *p* = .0013; *R^2^* = .04, *F* [1, 263] = 10.50, *p* = .0013) was underpinned by significant indirect effects via alexithymia alone (*b* = .01, CI: .00, .01) and via alexithymia and then anxiety sequentially (*b* = .00, CI: .00, .01). While in the formally-diagnosed group this total effect was non-significant (*b* = .01, *p* = .0544; *R^2^* = .02, *F* [1, 153] = 3.76, *p* = .0544), exactly the same two indirect effects remained: via alexithymia alone (*b* = .01, CI: .00, .02) and via alexithymia and then anxiety sequentially (*b* = .00, CI: .00, .01).

*DASS-Depression*

Interestingly, while autistic traits had been associated with DASS-21 total scores but not with DASS-Anxiety in the female groups, their association with DASS-Depression scores (path a^2^) was significant for the whole group (*b* = .14, *p* = .0067) and on the margins of significance in the formally-diagnosed group (*b* = .12, *p* = .0330). Alexithymia was strongly associated with DASS-Depression in both the whole female group (*b* = .25, *p* < .001; *R^2^* = .19, *F* [2, 262] = 30.14, *p* < .001) and the formally-diagnosed group (*b* = .35, *p* < .001; *R^2^* = .29, *F* [2, 152] = 31.05, *p* < .001).

As with DASS-21 total scores and DASS-Anxiety scores, DASS-Depression scores contributed significantly to the model predicting ED psychopathology in both the whole female group (*b* = .02, *p* = .0026; *R^2^* = .12, *F* [3, 261] = 12.14, p < .001) and the diagnosed group (*b* = .02, *p* = .0124; *R^2^* = .15, *F* [3, 151] = 9.20, *p* < .001); alexithymia also contributed significantly to both models (whole group: *b* = .02, *p* = .0043; diagnosed group: *b* = .02, *p* = .0092). In the whole group, while the direct relationship between autistic traits and ED symptomatology was non-significant (*b* = .00, *p* = .5802), a significant total effect (*b* = .04, *p* = .0013; *R^2^* = .04, *F* [1, 263] = 10.50, *p* = .0013) was underpinned by all three mediation effects (as had been observed with DASS-21 total scores): via alexithymia (*b* = .01, CI: .00, .02), via depression (*b* = .00, CI: 00., 01), and via alexithymia and via depression sequentially (*b* = .00, CI: .00, .01). For the diagnosed group, while neither the direct (*b* = -.00, *p* = .6912) or total effect of autistic traits on ED psychopathology (*b* = .01, *p* = .0544; *R^2^* = .02, *F* [1, 153] = 3.76, *p* = .0544) were significant, only two indirect effects were significant: an effect of autistic traits on ED symptomatology via alexithymia alone (*b* = .01, CI: 00., 02) and an effect via alexithymia and then depressive symptoms (*b* = .00, CI: .00, .01)
